# Biochar from Sugarcane Filtercake Reduces Soil CO_2_ Emissions Relative to Raw Residue and Improves Water Retention and Nutrient Availability in a Highly-Weathered Tropical Soil

**DOI:** 10.1371/journal.pone.0098523

**Published:** 2014-06-04

**Authors:** Angela Joy Eykelbosh, Mark S. Johnson, Edmar Santos de Queiroz, Higo José Dalmagro, Eduardo Guimarães Couto

**Affiliations:** 1 Institute for Resources, Environment, and Sustainability, University of British Columbia (UBC), Vancouver, British Columbia, Canada; 2 Department of Earth, Ocean, and Atmospheric Sciences, University of British Columbia (UBC), Vancouver, British Columbia, Canada; 3 Faculdade de Agronomia, Medicina Veterinária e Zootecnia (FAMEV), Universidade Federal de Mato Grosso, Cuiabaá, Mato Grosso, Brazil; 4 Instituto de Física, Universidade Federal de Mato Grosso, Cuiabá, Mato Grosso, Brazil; DOE Pacific Northwest National Laboratory, United States of America

## Abstract

In Brazil, the degradation of nutrient-poor Ferralsols limits productivity and drives agricultural expansion into pristine areas. However, returning agricultural residues to the soil in a stabilized form may offer opportunities for maintaining or improving soil quality, even under conditions that typically promote carbon loss. We examined the use of biochar made from filtercake (a byproduct of sugarcane processing) on the physicochemical properties of a cultivated tropical soil. Filtercake was pyrolyzed at 575°C for 3 h yielding a biochar with increased surface area and porosity compared to the raw filtercake. Filtercake biochar was primarily composed of aromatic carbon, with some residual cellulose and hemicellulose. In a three-week laboratory incubation, CO_2_ effluxes from a highly weathered Ferralsol soil amended with 5% biochar (dry weight, d.w.) were roughly four-fold higher than the soil-only control, but 23-fold lower than CO_2_ effluxes from soil amended with 5% (d.w.) raw filtercake. We also applied vinasse, a carbon-rich liquid waste from bioethanol production typically utilized as a fertilizer on sugarcane soils, to filtercake- and biochar-amended soils. Total CO_2_ efflux from the biochar-amended soil in response to vinasse application was only 5% of the efflux when vinasse was applied to soil amended with raw filtercake. Furthermore, mixtures of 5 or 10% biochar (d.w.) in this highly weathered tropical soil significantly increased water retention within the plant-available range and also improved nutrient availability. Accordingly, application of sugarcane filtercake as biochar, with or without vinasse application, may better satisfy soil management objectives than filtercake applied to soils in its raw form, and may help to build soil carbon stocks in sugarcane-cultivating regions.

## Introduction

The principal challenge for agricultural sustainability and feeding an ever-growing population is avoiding the degradation of soil, a vital but slowly renewable resource. In Brazil, economic and social drivers have come together to push agricultural expansion into pristine regions [1]. In addition to land use change in the Amazon and Atlantic forests, the savannah or Cerrado of Brazil's center-west has come under intense conversion, with an estimated 50% of this highly biodiverse region already converted to cropping and pasture, primarily within the last 40 years [2]. Conversion has increased the risk of soil degradation through soil organic carbon (SOC) loss, deteriorating soil structure, and increased risk of erosion [3], with knock-on effects for other ecosystem services.

Given that agriculture is central to Brazil's economic development, and that agricultural products from this region will help to feed and fuel the world, innovative agricultural technologies and practices are critically required to prevent soil degradation. Practices such as conservation agriculture have and will continue to play an important role [4], but supplementary strategies have also been proposed. One of these is to enhance soil storage of black carbon, which in natural soils has been associated with increased fertility, water retention, and more rapid incorporation or stabilization of new carbon inputs [5]–[7]. Black carbon in soil can be enhanced through amendment with man-made charcoal or *biochar* derived from the pyrolysis of waste biomass, through which relatively labile plant-fixed carbon is transformed into a highly recalcitrant char. Although most work to date has focused on temperate soils, studies incorporating biochar into tropical soils have shown evidence of enhanced soil quality, nutrient availability, and productivity, and decreased leaching losses and acidification [8]–[11].

Sugarcane cultivation in particular may benefit from this approach because the industry produces several pyrolyzable residues. These include bagasse (crushed cane stalks), cane trash (leaves and stalk tips removed during harvest), and filtercake, a sludge that is removed via filtration after the juice clarification step. Previous biochar studies on sugarcane residues have focused exclusively on bagasse, with positive results in terms of soil quality improvements and productivity [8], [9]. However, bagasse is a valuable by-product, both under current operations (*e.g.*, for cogeneration of heat and electricity in distilleries) and as a lignocellulosic feedstock for second-generation biofuels [12]. Thus, diverting bagasse for biochar production and soil amendment would come at the cost of other gains, and as such bagasse biochar may be less likely to be implemented as a strategy for soil and carbon management.

Filtercake, in contrast, is a heavy, nutrient-dense residue that is sometimes spread on fields as a fertilizer [13], although the high biological availability of its components likely leads to the rapid mineralization and potential leaching losses common in tropical soils [14]. Filtercake management is hampered by its high water content, which makes it costly to transport and difficult to apply, as well as its potential contribution to nutrient run-off and eutrophication when over-applied [12]. Thus, conversion of this highly labile residue into biochar may be an opportunity to turn a nuisance waste into a valuable soil amendment.

In this study, we developed a benchtop reactor to produce biochar from sugarcane filtercake for research purposes. Filtercake biochar was then characterized and its effects on nutrient availability and water retention analyzed. We also examined the effects of raw and pyrolyzed filtercake amendment on CO_2_ efflux in combination with vinasse, a carbon-rich effluent resulting from distillation that is similarly disposed of via soil application [13] and may itself affect carbon cycling through priming of native soil C [15]. Our analyses revealed several ways in which filtercake biochar is distinct from more commonly used woody and herbaceous biochars. Soil quality assays indicated that filtercake biochar may be useful as a soil carbon management tool with agronomic benefits for nutrient and water availability.

## Materials and Methods

### Material collection and biochar production

Permission to collect soil samples and organic residues (filtercake and vinasse) from private property was granted by the management of Usina Pantanal de Açúcar e Álcool Ltda, Jaciara, Mato Grosso, Brazil (15°55'28.11"S, 55°13'38.94"W, elevation 690 m). The soil was a red-yellow Ferralsol (FAO taxonomy) with a sandy clay loam texture (64% sand, 9% silt, 27% clay) and total carbon (C) and nitrogen (N) contents of 1.5% and 0.07% dry weight (d.w.), respectively. The mineral soil of the top 0–10 cm was collected and sieved to 2 mm. Vinasse was collected from canals close to the point of application and aliquots were frozen at −20°C until use. Vinasse total carbon concentration (1291 mg C L^−1^) was determined via combustion of the lyophilized solid residue to obtain % C (CHN-1110 elemental analyzer, Carlo Erba Instruments, Italy) and then multiplied by total solids (mg L^−1^).

Filtercake was collected fresh from the distillery and dried at 45°C for four days in a forced-air oven. The dried material was gently crushed and sieved to collect size fractions of 2–4 and 4–10 mm. Approximately 850 g of this material (50% from each size class) was pyrolyzed in a custom-made benchtop biochar reactor. The reactor consisted of a 10-×50-cm steel cylinder (diameter × length) closed on one end with a circular steel plate. The closed end was perforated with a 1/4″-brass male compression fitting to serve as an inlet for N_2_, which was used to purge the reactor of oxygen. The flanged open end was closed using a gasket made of high-temperature fibreglass cord and a steel plate held in place with a grooved circular clamp. The removable steel plate was perforated with two additional brass fittings, one for exhaust and a second to allow placement of a type-K thermocouple (Omega Engineering Inc., Stamford, CT) for monitoring internal temperature. These apertures were protected by a 1-mm stainless steel mesh at either end of the cylinder.

This assembly was mounted inside a large Linn Elektric muffle furnace with a programmable controller (KK 260 SO 1060; Linn High Therm GmbH, Germany). The slow pyrolysis program was as follows: slow heating to 575°C at a rate of 5–6°C min^−1^, holding at 575°C for 3 h, followed by slow cooling overnight to room temperature. The entire program was carried out under oxygen-limited conditions (N_2_ purge, 0.5–1 L min^−1^). The program was selected based on previous studies showing that this heating rate, pyrolysis temperature, and time were conducive to creating a biochar (from bagasse) retaining >500 mg C g^−1^ biochar [8], [9], [16]–[19].

### Biochar characterization

Biochar pH was determined by mixing 3 g of biochar in 27 g of water [17], followed by 30 min of intermittent shaking, 30 min of settling, and measurement with a handheld meter (HI 98121, Hanna Instruments, USA). Total C and total N contents were determined on a CHN analyzer (628 Series, LECO Corp., St. Joseph, MI).

In the carbohydrate analysis, filtercake and biochar samples were finely ground and extracted in hot acetone for 12 h to remove extractives (fats, resins, etc.). Next, 0.2 g of the dried extracted sample were incubated for 2 h in 3 mL of 72% H_2_SO_4_ (at 20°C), and then diluted to a final concentration of 4% H_2_SO_4_ and autoclaved at 121°C for 1 hour, followed by filtration. The filtered hydrolysate was then analyzed for carbohydrates via high-performance liquid chromatography, as described in detail by Huntley *et al*. [20].

Total and micropore surface area for both the initial feedstock (raw filtercake) and filtercake biochar were determined using the Brunauer–Emmett–Teller (BET) method [21], calculated from the N_2_ adsorption isotherm captured using an ASAP 2020 Physisorption analyzer (Micromeritics, Norcross, GA). Before analysis, samples were degassed at 90°C for 1 hour under a vacuum. For scanning electron microscopy (SEM), samples were sputter-coated with gold and analyzed using a Shimadzu SSX-550 microscope with an accelerating voltage of 15 kV. Finally, Raman spectroscopy was performed using a LabRAM HR system (HORIBA Jobin Yvon S.A.S., France). Spectra were captured using a 442-nm laser source, with a laser power on the sample surface of 1 mW and a 100× objective lens. Samples were acquired over an integration time of 60 s with two scans per sample. Data were analyzed using LabSpec 5 software (HORIBA Jobin Yvon S.A.S., France).

### Nutrient availability in soil mixtures

Macro- and micronutrient availability in biochar–soil mixtures were analyzed in solutions prepared at 1∶2.5 ratios (biochar–soil mixture∶distilled water). Analyses were carried out according to the standard soil methodologies published by the Empresa Brasileira de Pesquisa Agropecuaária (EMBRAPA) [22]. These included the following determinations: available P, Mehlich I extraction with spectrophotometric determination; K^+^ and Na^+^, Mehlich I extraction with flame photometry; Zn, Cu, Mn, diethylenetriaminepentaacetic (DTPA) extraction followed by atomic absorption; Fe, Mehlich I extraction with atomic absorption; Ca^2+^ and Mg^2+^, KCl (0.1 M) extraction followed by the complexometric titration method (EDTA method); Al^3+^, KCl (0.1 M) extraction followed by titration with NaOH; S, Ca_2_PO_4_ extraction followed by spectrophotometric determination; B, hot water extraction followed by colorimetric determination. Cation-exchange capacity (CEC) was calculated as the sum of exchangeable bases (Ca^2+^, Mg^2+^, and K^+^) plus potential acidity (H^+^ + Al^3+^).

### Water retention in soil-biochar mixtures

Water potential in soil–biochar mixtures (0, 5, or 10% biochar on a dry weight basis) were analyzed using a WP4C Dewpoint Potentiameter (Decagon Devices Inc., Pullman, WA). Briefly, each of the oven-dried soil–biochar treatments (*n* = 3 for each treatment) were divided into 5 sub-samples, which were then moistened with 0, 0.5, 1.0, 1.5, 2.0, or 3.0 mL of ultrapure water. These were allowed to equilibrate for at least 16 h, and then ∼0.5 g of each sub-sample were analyzed for soil water potential. Immediately afterward, each sub-sample was weighed, dried at 105°C overnight, and weighed again to determine soil water content. To facilitate the statistical analysis of replicates, water potential values (in pF) were plotted against binned soil water contents.

### CO_2_ efflux assay

The mineralization of raw *vs.* pyrolyzed filtercake was analyzed in the laboratory. Treatments were established as follows: soil alone (S); soil +5% filtercake (SF); soil +5% biochar (SB). To minimize disturbance, fresh, field-moist soil was sieved to 4 mm, roots were removed by hand, and then the soil was gently mixed to ensure a uniform composition; soil for all treatments was handled to an equal degree. Equal weights of these treatments were packed into incubation columns (10×15 cm, D×L; *n* = 6 each) and left open to the atmosphere in a temperature-monitored environment. CO_2_ efflux was determined on a daily basis for three weeks using a LI-COR 6400 XT apparatus (LI-COR, Lincoln, NE), which was fitted over the incubation column using a PVC sleeve and a foam rubber gasket. CO_2_ measurements were collected over three weeks under three conditions: week 1, field-moist (no added water); week 2, 30 mL water or vinasse (low dose); week 3, 60 mL water or vinasse (high dose). Vinasse and water were applied by slowly and uniformly dripping liquid over the soil surface using a pipette. CO_2_ fluxes were monitored over time and total CO_2_ flux and % C released was determined. Incubations were carried out at ambient temperature, ranging from 25–29°C during the night and 29–35°C during the day.

### Statistical analyses

Treatment effects were analyzed using one-way analysis of variance (ANOVA) with *post-hoc* Tukey tests to detect significant differences among means. All data are presented as the mean ± the standard error (SE). A *p* value <0.05 was considered significant. All statistical analyses were carried out in R v.3.0.1 [23] using Rcmdr [24].

## Results

### Filtercake biochar production and characterization

The pyrolysis of raw filtercake yielded what appeared to be a fully pyrolyzed product (biochar yield  = 36% dry feedstock weight), as well as an unquantified amount of bio-oil. Filtercake biochar was alkaline in water (pH = 9.85±0.08) and contained 36.7% C dry weight, and 1.3% N dry weight (C∶N, 28), whereas raw filtercake contained 37.4% C and 1.2% N by weight (C∶N, 32). Pyrolysis greatly depleted but did not wholly eliminate carbohydrates. Raw filtercake contained 18% cellulose (d.w.) and 11.9% hemicellulose, as well as 32% lignin; filtercake biochar retained 2.7% cellulose and 3.1% hemicellulose (lignin not determined).

**Figure 1:**
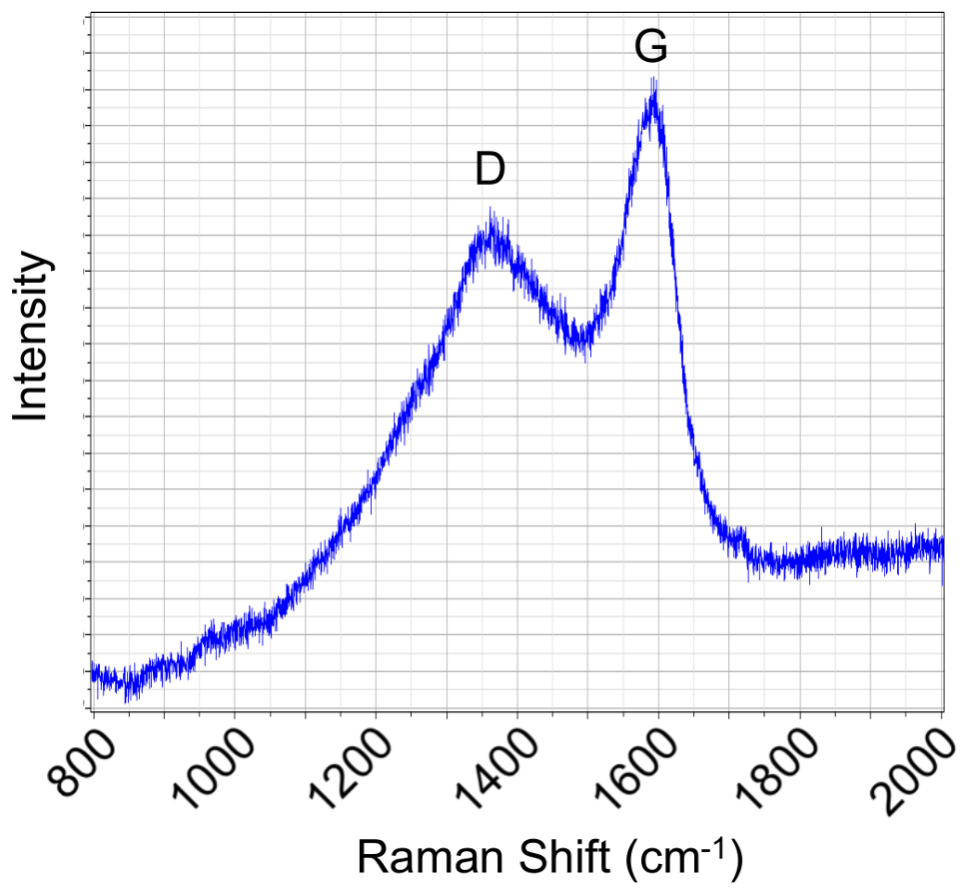
Raman spectrograph of filtercake biochar. D, defect band (1360 cm^−1^); G, graphite band (1590 cm^−1^).

The biochar was analyzed by Raman spectroscopy, which revealed a characteristic two-peak spectrum (**Fig. 1**) typically displayed by carbonaceous materials with a core aromatic structure, including biochars [*e.g*., 25]. The first peak, originally designated the D or *defect* band (1360 cm^−1^), was initially associated with disordered graphite structure, but has since been associated with benzene rings [26]. The G or *graphite* band (1590 cm^−1^) is characteristic of graphite; however, in the case of chars, previous work showing the lack of detectable graphitic C via X-ray diffraction led authors to attribute this peak instead to quadrant aromatic ring breathing [27]. These results suggest that the filtercake biochar produced is primarily composed of aromatic C.

**Figure 2:**
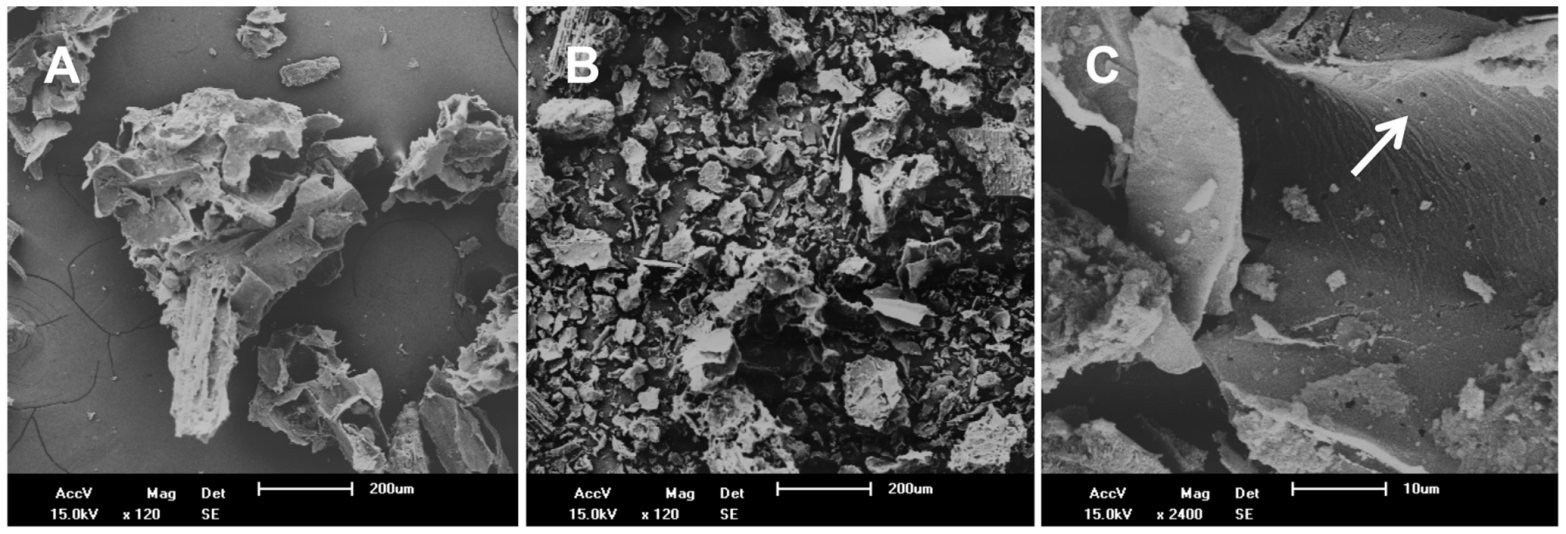
Scanning electron micrographs of raw filtercake and biochar prepared via slow pyrolysis at 575°C. (A) Raw sugarcane filtercake at a magnification of 120×. (B,C) Filtercake biochar at magnifications of 120× and 2400×, respectively. White arrow indicates micropore formation.

Finally, N_2_-BET surface analysis revealed that slow pyrolysis increased the specific surface area of raw filtercake from 0.38 m^2^ g^−1^ to 26.3 m^2^ g^−1^ in the charred product. Analysis of deBoer t-plot micropore area revealed that only approximately 5.5 m^2^ g^−1^ of this surface area was contained within pores <2 nm in diameter. Scanning electron microscopy of both the raw filtercake and pyrolyzed product revealed differences in particle size due to fracturing during pyrolysis (**Fig. 2A,B**). Biochar particles were angular with large irregular macropores. At high magnification (2400×), some biochar particles showed scant formation of pores <1 µm in diameter (**Fig. 2C**).

### Effect on soil quality parameters and nutrient extractability

Mixing an air-dried dystrophic red-yellow Ferralsol with increasing amounts of filtercake biochar led to increases in soil pH, cation exchange capacity, and carbon, nitrogen, and nutrient availability for increasing levels of biochar (**Table 1**). Even the low dose of 1.25% biochar markedly increased the availability of P, K, and Ca. Among micronutrients, the addition of biochar most strongly affected the extractability of Fe, Mn, and Zn, although these effects were only apparent at a treatment of 5%. This increase in extractable nutrients may be due to a direct contribution from the biochar amendment, as well as increases in nutrient availability due to the pH change resulting from the addition of biochar.

**Table 1:** Physicochemical characterization of soil–biochar mixtures.

*Parameters*	*--------------------------- % Biochar (by weight) ---------------------------*			
	*0%*	*1.25%*	*2.5%*	*5%*
pH (H2O)	6.13±0.03, d	6.70±0.06, c	7.03±0.03, b	7.40±0.06, a
CEC	8.0±0.2, b	9.0±0.2, b	10.5±0.2, a	11.6±0.4, a
% C	1.48±0.06, b	—	—	2.65±0.03, a
% N	0.07±0.01, a	—	—	0.08±0.01, a
***Macronutrients***				
P	2.5±1.0, b	107.8±5.8, a	146.0±22.8, a	151.6±17.8, a
K	46.7±0.7, d	114.3±3.2, c	164.3±3.5, b	243.0±10.8, a
Ca	3.1±0.1, c	4.9±0.1, b	6.1±0.1, a	6.7±0.3, a
Mg	1.5±0.1, c	2.0±0.1, c	2.7±0.2, b	3.5±0.2, a
S	13.1±0.7, a	13.9±0.1, a	13.1±0.1, a	13.2±0.3, a
***Micronutrients***				
B	0.26±0.02, a	0.27±0.02, a	0.29±0.01, a	0.28±0.01, a
Fe	115.0±12.8, b	173.7±14.5, ab	195.7±18.8, ab	223.7±29.6, a
Mn	7.1±0.9, b	8.0±0.9, b	10.1±0.7, ab	12.9±0.5, a
Zn	2.8±0.6, b	3.7±0.6, b	4.3±0.2, b	6.4±0.1, a

All statistical comparisons were performed using one-way analysis of variance with a *post hoc* Tukey test. Data represent the mean ± SE. P and K values are given in mg dm^−3^. Remaining nutrient values and CEC and all other nutrients are given in cmol_c_ dm^−3^.

### Water retention

**Figure 3:**
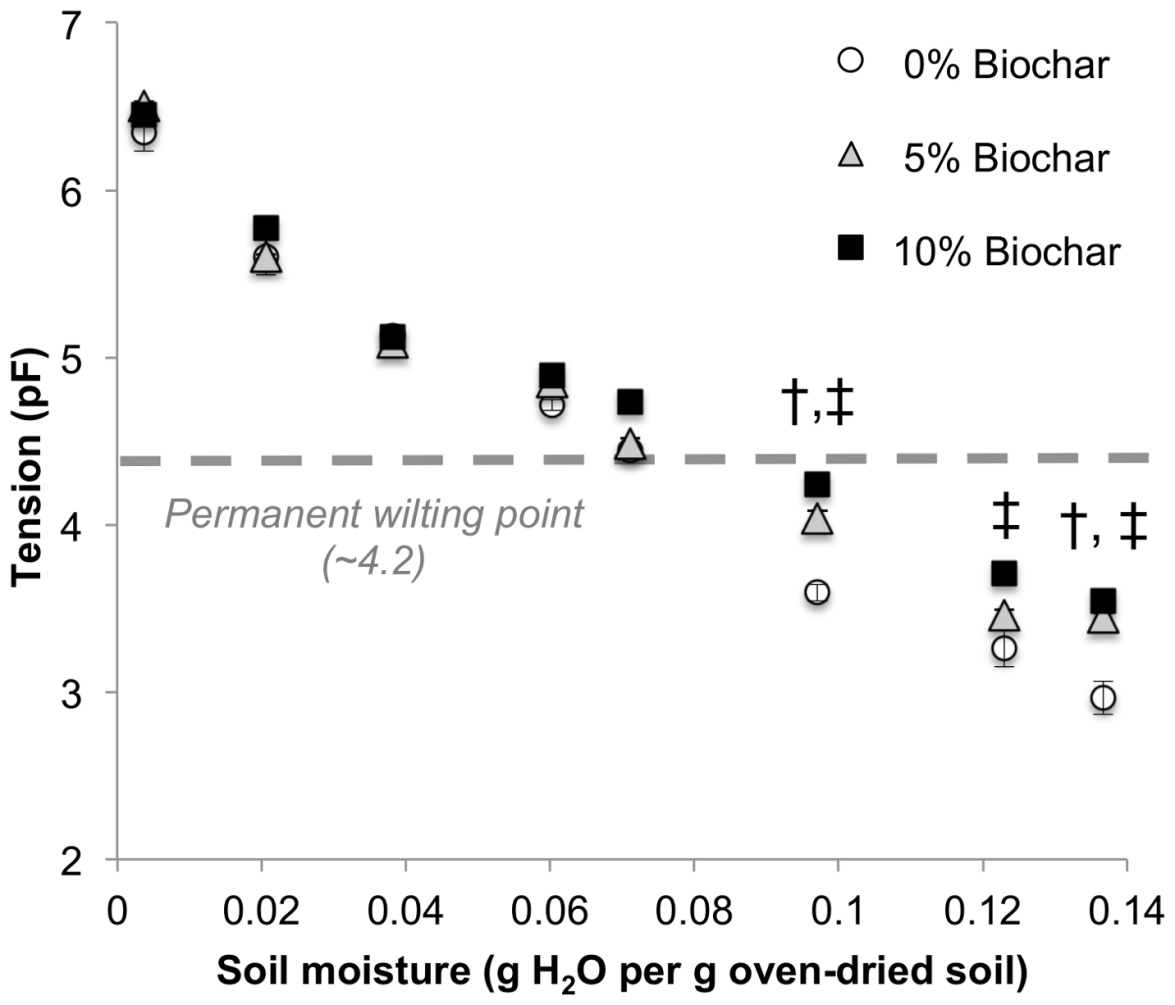
Soil matrix potential and moisture content in soils containing 0, 5, or 10% filtercake biochar. Treatments were compared using one-way ANOVA with a *post hoc* Tukey test for samples in each soil moisture bin. Data represent the mean ± SE. Note that significant differences were observed only below the permanent wilting point (dashed line). †, significant with respect to 5% biochar treatment; ‡, significant with respect to 10% biochar, *p = *0.05.

The effect of biochar on soil water retention was examined by generating matric potential–soil moisture curves for soil alone and soil mixed with 5 or 10% biochar. The addition of 5 or 10% biochar led to a dose-dependent increase in soil matric potential at a given soil moisture content; importantly, this effect was only observed within the range of soil water potentials coinciding with plant-available water (pF<4.18), and grew more pronounced at the wetter end of the curve (**Fig. 3**). These data suggest that filtercake biochar increased the availability of pores that release water at potentials less than pF = 4.18 (pores >0.2 µm in diameter) [28]. This is consistent with our N_2_-BET surface area analysis, which found that this filtercake biochar showed very little surface area in pores <0.2 µm in diameter, which is desirable in terms of retaining plant-available water.

### Short-term CO_2_ effluxes

**Figure 4:**
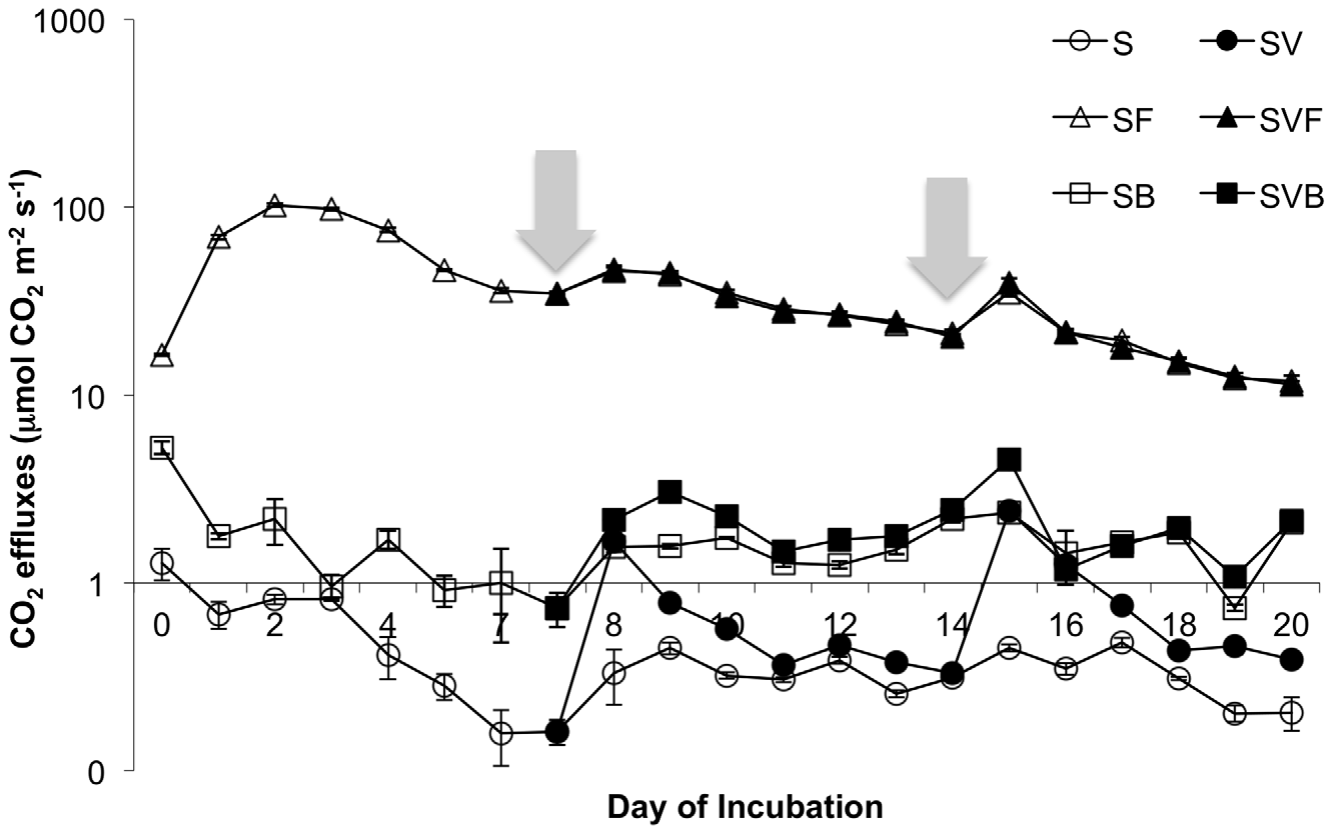
CO_2_ effluxes from soil treated with 5% raw filtercake or 5% biochar under field-moist conditions. Data points represent the mean treatment value ± SE at each time point. Note that data are plotted on a logarithmic scale. Water (open symbols) or vinasse (solid symbols) was added on the days indicated by a gray arrow; 30 mL were added at the beginning of week 2 and 60 mL at the beginning of week 3. S, soil; SV, soil with vinasse; SF, soil with 5% filtercake (d.w.); SVF, soil with 5% filtercake and vinasse; SVB, soil with 5% biochar and vinasse; SB, soil with 5% biochar.

**Figure 5:**
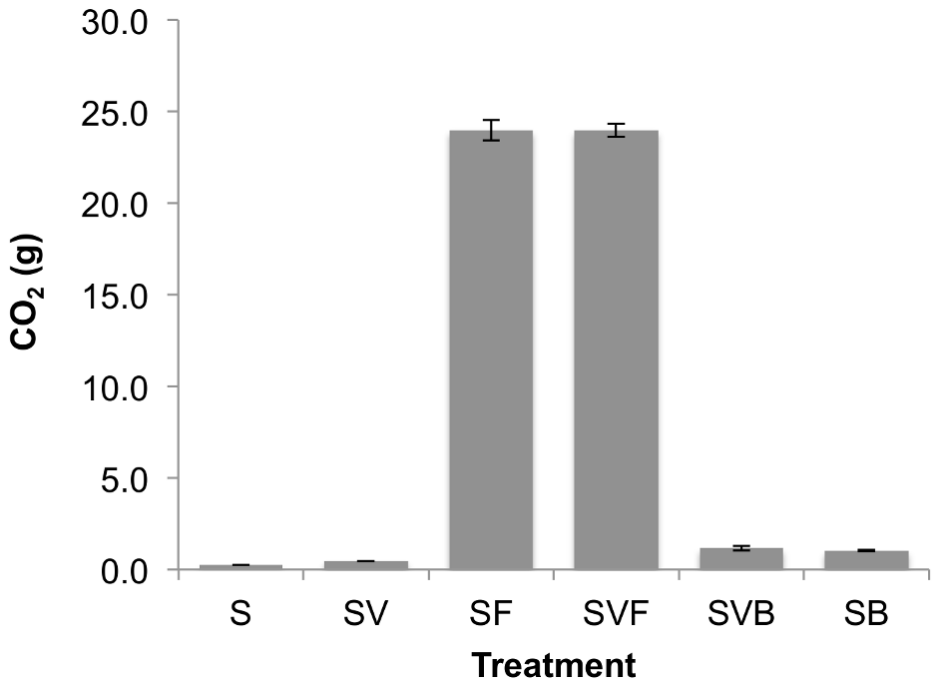
Total CO_2_ effluxes from soil and soil amended with filtercake, biochar and/or vinasse. Treatments included the control (soil, S), soil amended vinasse (SV), soil with 5% filtercake (d.w.) (SF), soil amended with filtercake and vinasse (SVF), soil amended with 5% biochar (d.w.) produced from filtercake (SB), and soil amended with biochar and vinasse (SVB). These data show the strong effect of applying filtercake as biochar on reducing CO_2_ effluxes (*e.g.*, SF *vs*. SB and SFV *vs*. SBV).

To examine the effect of sugarcane residues on soil respiration and carbon utilization, field-moist soil was mixed with filtercake or biochar and incubated for three weeks. During the first 7 days, a large initial efflux of CO_2_ was observed for soil amended with filtercake (SF), which peaked after two days; the soil plus biochar (SB) and soil-only treatment showed a more muted response that began to decrease immediately (**Fig. 4**). Respiration was also moisture-limited, as evidenced by brief increases in CO_2_ efflux that accompanied water or vinasse addition (gray arrows in **Fig. 4**). Among treatment groups, soil-only (S) incubations showed the lowest mean efflux, whereas biochar incubations (SB) were slightly but consistently higher over time. Vinasse application increased CO_2_ efflux in the SV and SVB incubations relative to the S and SB controls, respectively. After calculating total g CO_2_ emissions over the entire experimental period, we found that the addition of raw filtercake to soil (SF) led to a 100-fold increase in g CO_2_ emitted relative to the unamended soil (S) control. However, this large increase was greatly ameliorated by applying the filtercake as a pyrolyzed product (**Fig. 5**).

To further investigate the effects of biochar and vinasse on soil C emissions, data were analyzed with respect to percent C lost from the treatment, based on a calculated estimate of the amount of soil carbon plus amendment carbon added. This revealed that filtercake treatments lost a much greater percentage of total C (SF, 16.3±0.4% C), most likely due to a much larger proportion of filtercake feedstock that was enriched in cellulose and hemicellulose (approx. 55% of total C). Vinasse application with filtercake had no additional effect (SVF 16.2±0.2%), perhaps because the soil had reached its respiratory maximum for the available amount of water. Because the powerful effect of filtercake addition greatly skewed the data set, we examined the differences amongst the S, SV, SVB, and SB treatments separately using oneway ANOVA. This revealed that although total carbon loss from soil-only incubations was low (S, 0.35±0.02% of total C present), vinasse addition did significantly increase overall % C lost (SV, 0.70±0.02%, *p* = 0.003; **Table 2**), as would be expected given this highly labile substrate. Biochar addition alone (SB) also led to a minor increase in the total C lost (0.69±0.15%, *p* = 0.003 with respect to S), perhaps due to increased aeration as a result of a slight decrease in soil bulk density (S, 1.08 g cm^−3^; SB, 1.04 g cm^−3^, *p*<0.001). Vinasse addition did not augment this effect (SVB, 0.78±0.04%) compared to biochar alone (SB, 0.69±0.15%, *p* = 0.54).

**Table 2:** Total CO_2_ emitted and percentage of total carbon lost after three weeks of incubation.

*Treatments*	*CO_2_ emitted*	*Total C content*	*Total C lost*
	(g CO_2_)	(g C)	(%)
S	0.24±0.01, a	18.3	0.35±0.01, a
SV	0.47±0.01, a	18.4	0.70±0.01, b
SVB	1.17±0.13, b	40.7	0.78±0.08, b
SB	1.04±0.03, b	40.6	0.69±0.02, b

To further probe differences among the S, SV, SVB, and SB treatments, total CO_2_ emitted and % C lost were compared via one-way ANOVA. Data represent the mean ± SE. Total C content refers to the sum of initial carbon present in the soil plus C added through amendments. Letters indicate significant differences between treatments as determined using Tukey HSD test.

S, soil; SV, soil with vinasse; SVB, soil with 5% biochar and vinasse; SB, soil with 5% biochar. S and SB received water at the beginning of week 2 (30 mL) and week 3 (60 mL). SV and SVB received the same amount of vinasse.

## Discussion

It is frequently noted in the biochar literature that feedstock and pyrolysis conditions are the determining factors in final char characteristics [9], [16], [17], [29]. Here, we compare filtercake biochar with its related product, bagasse biochar as reported in the literature, as well as other feedstocks, and compare these in terms of their agronomic benefits.

Biochars are often proposed as a means to enhance soil black carbon stocks. In natural soils, black carbon plays an important role as a geosorbent and microhabitat [30], a modifier of soil chemistry and carbon cycling [6], [7], and as a slowly available C source for microbial activity [31]. In undisturbed Cerrado ecosystems, black carbon produced through natural fires makes up a small but stable pool within the total soil carbon stock; however, this stock is depleted after disturbance [32].

In this study, filtercake biochar produced under the described pyrolysis conditions falls under the category of a low-carbon char, according to the biochar classification proposed by Joseph *et al*. [29]. This is somewhat surprising given that our feedstock was relatively rich in lignin (32%) compared to softwoods and other non-woody residues [33]. Previous work has shown that lignin has a higher char yield (C in feedstock retained in char, 49%) compared to cellulose (19%) or hemicellulose (23.5%) after pyrolysis at 800°C [34], and feedstocks rich in lignin often produce chars with higher % C [17], [35]. For example, bagasse with an initial lignin content of approximately 20% [12], [33], produces chars in the range of 63–84% C at 600°C [8], [9], [18]. Thus, the filtercake char described here had less C than might be expected for a lignin-rich material pyrolyzed near to 600°C. Because filtercake has been subjected to fermentation, the depletion of biodegradable non-lignin C may account for both the overall low C yield in the biochar product as well as lignin enrichment in the filtercake.

Nevertheless, filtercake biochar may be very valuable as an agricultural amendment. Regarding its effects on nutrients, treatments as a low as 1.25% biochar (d.w.) significantly increased P, K, and Ca availability and treatment at 5% increased micronutrient availability (**Table 2**) in a dystrophic red-yellow Ferralsol. Furthermore, in an agricultural context, the presence of a small amount of non-pyrolyzed carbohydrates can be viewed as a benefit as these compounds represent a source of bioavailable nutrients and energy for the soil microbial community.

Surface area is a key physical characteristic of biochars because it indirectly indicates a char's ability to retain water as well as dissolved nutrients and low-molecular weight carbon compounds through pore filling [36]. In this study, we noted that filtercake biochar pyrolyzed at 575°C had a high N_2_-BET surface area (26 m^2^ g^−1^) compared to the raw filtercake feedstock (0.38 m^2^ g^−1^), but a relatively low N_2_-BET surface area compared to bagasse pyrolyzed at close to the same temperature (218 m^2^ g^−1^ at 600°C) [9]. Further SEM analyses revealed highly angular, macroporous particles (Fig. 2b), demonstrating why biochar addition decreased bulk density and increased porosity in treated soils. Pore formation depends largely on production parameters and original biomass structure [37]. Although some remnants of the original plant vasculature were observed through SEM (data not shown), filtercake biochar overall lacked the regular, highly porous structure observed in bagasse biochar made at near the same temperature [17], [18]. Because filtercake is subject to maceration and fermentation, destruction of the plant's vascular structure may have contributed to relatively low total surface area.

Nevertheless, even this small increase in total surface area and porosity over the raw feedstock may underlie the increase in soil matrix potential in soil-biochar mixtures. Our results showed that biochar dose-dependently increased the soil matric potential for a given quantity of water added, indicating that water was held more tightly, and this effect was significant within the range of plant available water (pF<4.18). This work is consistent with numerous previous studies showing that biochar increases plant-available water, especially in sandy soils similar to the one examined here [9], [38]–[40]. Given that modeling studies have predicted a warmer, drier future for this region of Brazil [41], utilizing filtercake as biochar to increase plant-available water may be a useful climate adaptation strategy.

Biochar is also posited as a possible climate mitigation strategy, based on the assumption that aromatic carbon within biochar is highly resistant to microbial attack, and may therefore increase carbon sequestration [42]. However, the effect of biochar on soil respiration and carbon turnover is complex. Although some studies have reported that biochar suppresses or has no effect on soil respiration [16], [43], others report a transient or sustained increase in CO_2_ efflux [16] and microbial biomass [44] over the study periods, suggesting a biological response. We also noted a small but sustained increased in CO_2_ efflux from biochar-amended soils relative to the untreated control, and that biochar-treated soils lost a minor, though overall greater percentage of total C compared to the unamended soil (**Table 2**). This may indicate a priming effect, which is defined as an increase in the turnover of existing soil organic carbon in response to carbon addition that is mediated by soil microbes [15]; these priming effects occur in many systems and their ecological consequences are not well understood. Alternatively, increased CO_2_ efflux in the presence of biochar may occur through an abiotic mechanism, such as oxidation or off-gassing of CO_2_, which in some studies have accounted for >50% of CO_2_ emitted from amended soils [45], [46]. Regardless, the amount of C lost via abiotic or biotic mechanisms seems to be dependent on biochar production temperature; chars produced at higher temperature with lower remnant labile or volatile C produced a smaller or suppressed efflux [47]. Thus, although the filtercake biochar used here was produced at a relatively high temperature, the residual cellulose and hemicellulose detected in the carbohydrate analysis may have been responsible for elevated soil CO_2_ effluxes.

### Use of raw vs. pyrolyzed filtercake in sugarcane cultivation

Recent lifecycle assessments examining the contribution of soil organic carbon to total carbon emissions from biofuel systems have generated new appreciation for crop residue management and its role in keeping carbon in the soil [48]. Returning sugarcane residues to the field is promoted as a means to re-build C stocks and re-capture valuable nutrients [13], [49], [50]. However, residue application also provokes transient increases in N_2_O and CO_2_ emissions [51], [52], suggesting that this biomass undergoes relatively rapid mineralization and may contribute to further negative effects. Perhaps because of this, sugarcane cultivation areas in Brazil often show low to no recovery of soil C relative to the pre-cultivated baseline, despite large annual inputs from these residues [53]–[55].

To mitigate SOC degradation, several previous authors have called for the integration of biochar science into biofuel cultivation and crop management [42], [56]. The objective of this paper was to reconsider the waste product, filtercake, and gauge its potential merits as a biochar soil amendment through chemical characterization and a panel of assays assessing the most critical agronomic benefits of a biochar. Filtercake biochar showed benefits in terms of increasing soil pH, CEC, nutrient availability, and water retention. These parameters are directly relevant to exploratory studies investigating the effect of biochar amendment on sugarcane productivity via the use of biophysical models (*e.g.*, APSIM-sugarcane [57]). Furthermore, raw filtercake led to a large increase in soil respiration and a greater percentage of initial carbon lost compared to biochar treatment, likely due to the immediate utilization of labile sugars present in the filtercake. Although this was a very short-term study, from which it is not possible to extrapolate long-term biochar stability, it demonstrates the large difference in biodegradability between filtercake in its raw *vs.* pyrolyzed forms.

As such, the use of raw filtercake may represent a lost opportunity for soil improvement in this region, where a hot, semi-humid climate and nutrient-poor, leachable, acidic soils severely limit plant productivity and increase management costs [58]. In contrast, applying filtercake as biochar may slow the loss of organic carbon from the field, and contribute to a liming effect, increased CEC, water retention, nutrient availability, and activation of the soil microbial community, all of which are positive indicators of soil quality. Thus, our data suggest that soil improvement objectives on sugarcane plantations might be better served by applying filtercake as biochar, rather than the raw material. Biochar implementation may also have economic benefits, as high-temperature pyrolysis is exothermic and self-sustaining through the production of syn-gas; it also generates saleable bioenergy products (bio-oil and biochar itself) and shows potential as a tradable GHG emission-reducing soil amendment [59]. However, further studies are required to determine the long-term stability of this biochar and how this product might influence lifecycle C emissions, as well as its effects on plant productivity and the feasibility of incorporating pyrolysis bioenergy and biochar into plant operations.

In summary, physical and chemical characterization revealed that the novel filtercake biochar described here has significant benefits in terms of increased CEC, pH, nutrient availability, and water retention, indicating that this product may be beneficial as a soil amendment. Importantly, we also found that filtercake applied as biochar greatly reduced soil CO_2_ effluxes compared to the application of raw filtercake, without wholly eliminating microbial activity. These findings open the way to further studies examining the use and stability of filtercake biochar in the field, and demonstrate in principle how organic waste management on sugarcane plantations could be modified to improve soil quality and reduce CO_2_ effluxes from cultivated areas.

## Supporting Information

Table S1 **Raw data for physicochemical characterization of soil-biochar mixtures presented in** **Table 1** **.**
(CSV)

Table S2 **Raw data for calculating soil matrix potential against moisture content in soils containing 0, 5, or 10% filtercake biochar, as presented in** **Figure 3** **.** Binned water content values represent the average true water content determined gravimetrically for all samples (0, 5, and 10% biochar) for a set of samples to which a specific amount of water was added before analysis. This allowed statistical comparison of tension (pF) values for the treatment groups for a given moisture content.
(CSV)

Table S3 **Raw data for daily and total CO_2_ effluxes presented.** The first data set represents daily CO_2_ fluxes obtained from incubation columns over a three-week period, as presented in Figure 4. The second data set represents CO_2_ emitted (µmol m^−2^) in the preceding 24 hours. Where values were missing in the first data set, CO_2_ emitted was calculated as the area under the trace based on the next available timepoint assuming a linear increase or decrease between the two time points. Daily CO_2_ effluxes were summed to obtain total efflux, which was then converted into g CO_2_, for which treatment means were compared in Figure 5. Next, to further probe differences among the low-emitting treatments, the S, SV, SVB, and SB data were analyzed via oneway ANOVA for both g CO_2_ emitted and total percent C lost. Total percent C lost refers to g CO_2_-C emitted divided by the total amount of carbon present in the incubation (soil carbon plus added biochar/vinasse/filtercake carbon). The treatment means for g CO_2_ emitted and % C lost for the S, SV, SVB, and SB treatments are presented in Table 2.
(CSV)
